# Seven Letters and Replies

**Published:** 1874-04

**Authors:** 


### Our Correspondence.

### Letters Exposing Humbugs and Swindlers,

### With other Communications of Interest to the People.

DEAR BISTOURY:

The article quoted in your January number on "breaking in" boots and shoes, is quite sensible. I remember some years ago certain shoemakers had a plan for making easy boots. They would set your foot on a piece of paper, mark around it with a pencil, so as to get the exact form, and then carefully make the boot *some other shape.* Is that done in Elmira?

NIEMAND.

*Ich wissen nicht.*

---

NEWBURYPORT, MASS., July 6th.

DR. UP DE GRAFF:

DEAR SIR:—Enclosed find fifty cents, the amount of one year's subscription to the BISTOURY. 'Though I take a great many medical journals, I could not do without the BISTOURY, either in my private practice or in the retirement of my study.

Respectfully yours, E. P. H.

We take considerable pride in printing the above letter, as it is the opinion of one of the most distinguished physicians of Massachusetts, one whose coutributions to medical science are frequently seen in our principal medical journals.

---

DENVER, COL., Jan. 15, 1874.

DR. UP DE GRAFF:

* * * * The atmosphere here is so clear, I can see over one hundred miles. I should judge there to be about three thousand sick people in this city, and more coming. People die here every day, from consumption,—those who have come expecting a cure, but who have waited too long. The climate seems to be a "sure cure" for asthma, provided such invalids *remain* here. Have conversed with a great many very bad cases, *all* of whom are entirely free from the complaint *as long as they remain here.* When they return east again, the old symptoms return too.

I am feeling much better than when I left Elmira, and hope by a continued residence here to entirely recover from my lung difficulty. J. F. B.

Mr. B. is threatened with consumption. We advised him to go to Colorado, and the above is his message from there, or so much of it as would be interesting to our readers.

---

MONROE, N. Y., Feb. 12, 1874.

DR. UP DE GRAFF :

A man by the name of Wm. Hallock, of this place, while deranged, visited Elmira. He says he has at your place an overcoat and overshoes. Will you befriend a poor unfortunate man so far as to have them expressed to him at this place. T. Mc G.

Give yourself no uneasiness over William's condition. We would not recommend a residence for him in an insane asylum at the public expense. Such an arrangement would be too much in accordance with his ideas of complete comfort. William is not crazy—not he. He 's lazy, that 's all. You see, he does not like work,—says it hurts him so. He would much prefer going to an asylum, or to jail, for that matter, could he be assured of immunity from labor. In relation to his clothes, he left none here, any more than was he robbed of that $75, the loss of which he so much deplored when an inmate of the Institute, at our expense. Believe me, William is a fraud,—a shallow one, too, who needs only to be kicked out of doors and compelled to work for his own support.

---

MEMPHIS, TENN., Feb. 20, 1874.

DEAR BISTOURY :

If I did not have in mind that case of the itching scalp, and the colt case of long ago, I should be glad to ask for some information myself. May I venture without risk of appearing ridiculous ?

I am troubled with catarrh,—have been for years, until my health is failing by reason of it. Our doctors give me no assurances that it can be cured, so I am using some patent medicines recommended for its cure. I find everything I use makes me worse, until now I am entirely incapacited for business. What *can* I do ? L. R.

Quit using patent medicines. They are not intended to cure *anything*. They are made to *sell*,—that and nothing more. If your physicians are not provided with suitable apparatus for treating catarrhal affections, go to the nearest specialist, of good repute, and place yourself under his care. Catarrh *can* be cured, but once cured, requires careful watching and attention, during a year or more, to *keep* it well.

---

SYRACUSE, N. Y., March 1, 1874.

THAD. S. UP DE GRAFF, M. D.:

Will you please reply to the following questions in your "Correspondence," in the next pamphlet ? What is good to lull an extremely *nervous* and *sleepless* person to sleep, —one who lays awake many nights, and gets no relief ? And is there any remedy for *freckles*, except the unhealthy one of staying in doors ? By answering you will greatly oblige me. MRS. E. K. W.

There must be some cause for your wakefulness. Consult your family physician with reference to the matter. Pure *chloral hydrate*, twenty grains, dissolved in one ounce of cinnamon water, and taken at bed time, will insure a good night's rest, and do you no harm in the taking of it. The following would be the proper formula to hand to your druggist :

℞

Chloral hydrate, ʒ j.

Aqua cinnamonum, f. ℥ iv.—M.

Sig :—Take a large tablespoonful at bedtime. A larger dose can be taken if the desired effect is not secured. Look in back numbers of BISTOURY for recipes for freckles. Quite a number of formulæ have been given.

---

NEW YORK, Nov. 21, 1873.

MR. PUBLISHER :

If you will publish the above advertisement in your paper for six months, at your lowest rates and send me the bill payable in advance with proof that the advertisement is being published, I will pay the amount.

M. HOURE.

The advertisement referred to is to the effect that the above mentioned M. Houre, is a dealer and trafficer in divorces. That is to say, he secures divorces for *any* one who chooses to apply, "without publicity." Of course, this can only be accomplished through deception and swindling, in a manner that has been fully exposed in these columns before. We are not in need of such patronage for our columns, and *again* inform Mr. Houre that his advertisement cannot be printed in the BISTOURY at *any* price.

